# Effects of Far-Infrared Radiation Drying on Starch Digestibility and the Content of Bioactive Compounds in Differently Pigmented Rice Varieties

**DOI:** 10.3390/foods11244079

**Published:** 2022-12-16

**Authors:** Jiranan Ratseewo, Frederick Jame Warren, Naret Meeso, Sirithon Siriamornpun

**Affiliations:** 1 Division of Food Science and Technology, Faculty of Liberal Arts and Science, Sisaket Rajabhat University, Sisaket 33000, Thailand; 2 Quadram Institute Biosciences, Food and Health, Norwich Research Park, Norwich NR47 UA, UK; 3 Research Unit of Drying Technology for Agricultural Products, Faculty of Engineering, Mahasarakham University, Kantarawichai, Maha Sarakham 44150, Thailand; 4 Research Unit of Thai Food Innovation, Department of Food Technology and Nutrition, Mahasarakham University, Kantarawichai, Maha Sarakham 44150, Thailand

**Keywords:** pigmented rice, starch digestion, phenolics, anthocyanins, far-infrared radiation

## Abstract

Far infrared radiation (FIR) was applied to six rice varieties with different coloring of the pericarp (purple, red or non-pigment). Changes were determined in amylose content, in gelatinization parameters, in the content of bioactive compounds, in antioxidant activity and in the in vitro digestibility of pigmented rice as affected by FIR. The highest contents of amylose, total phenolic (TPC), total flavonoid (TFC) and total anthocyanins (TAC) were found in the purple and red varieties. Overall, FIR increased TPC, TFC and TAC, including antioxidant capacity. Quercetin and apigenin contents were increased while rutin and myricetin decreased significantly (*p* < 0.05) in all FIR-dried samples. Dephinidin, cyanidin-3-glucosides and pelargonidin increased after FIR treatment. Mostly, FIR-treated samples were found to have greater gelatinization enthalpy, compared with unheated rice samples. FIR-dried rice showed lower starch digestibility (25–40%) than unheated rice. This research suggested that the specific genotype of rice had the greatest influence on amylose content in pigmented rice, while FIR drying had no further effect. Our results suggest that FIR could enhance the content of the bioactive compounds capable of inhibiting α-amylase, thereby lowering starch digestibility. Hence, FIR may be considered as an appropriate drying method for pigmented rice regarding health benefits.

## 1. Introduction

Rice (*Oryza sativa* L.) is an important cereal crop and is widely consumed in many countries of the world. Mostly, white rice (non-pigmented or normal rice) is widely consumed as a staple food in Asia. However, over the past decade, pigmented rice (purple, black, red coloring, etc.) has been gaining more attention from consumers because of its perceived health benefits. The pigments on the seed coat have been implicated for enhancing the function of the immune system. Furthermore, pigments of the rice-bran fractions may inhibit allergic reactions in vitro [1]. The various types of bioactive substances in rice are generally found in the germ, bran and outermost aleurone layers of the grain, and they include colored pericarp layers, depending on the varieties of rice [2,3,4].

The postprandial blood-glucose-raising potential [5] of rice has been established as an indicator of starch digestibility, thus enabling the selection of foods containing abundant amounts of carbohydrates, therefore avoiding the accrued risk of non-communicable diseases, such as type-2 diabetes, cardiovascular disease and obesity, which may be related to the continuous consumption of high-starch digestion or high glycemic index (GI) foods [5]. The pigmented pericarp of rice comprises various phytochemicals, mainly phenolic compounds and anthocyanins [6,7]. These compounds have been reported to possess anti-oxidative effects and are capable of reducing the intensity of oxidative free radicals [1,8]. Many studies have indicated the significant biological value of compounds such as antioxidants and compounds with anti-cancer, anti-viral, or anti-inflammatory activities, thus offering health-promoting properties [1,9,10]. Furthermore, there has been a number of studies reporting that the starch digestibility of pigmented rice is less than that of normal rice. Such pigmented varieties include pigmented Indian rice [11] and pigmented Thai rice varieties [12,13]. Moreover, researchers reported that phenolic compounds, including anthocyanins, are potent inhibitors of α-amylase and α-glucosidase, these being important enzymes associated with carbohydrate digestion and sugar absorption, thus related to diabetes mellitus. They can be supportive for hyperglycemia control and type-2 diabetes-mellitus treatment through lowering blood-glucose levels [14,15,16,17].

Nevertheless, alterations in the quantity and composition of these compounds may be affected by drying methods currently used in the cereal-crops industry. The drying process plays a major role in decreasing moisture content for safe storage. Far-infrared radiation (FIR) has been applied for drying biomaterials, including agricultural products used for food [18,19]. FIR drying involves heat from rays transferred smoothly to the centre of the material being dried, thus causing vibration of the bonds linking molecules or components within the material, without (or only slightly) decomposing molecules on the material’s surface. Recently, several studies have reported changes in physical properties, in bioactive compounds and in the antioxidant activity of agricultural products as a result of FIR treatment. Examples include water extraction from peanut hulls [20], Kaprow leaves [19], rice bran [18] and pigmented rice in Thailand [4]. In addition, Tangkhawanit et al. [21] have reported that the effects of FIR-HA drying increase the content of bioactive components, such as total phenolic, flavonoid and isoflavone in whole soybean and soy residue, thereby providing improved inhibition of the enzyme in soymilk residue extracts and lowering starch digestion. From our previous studies, we had reported that the starch digestibly of six varieties of pigmented rice was lower than that for normal rice. These changes were associated with higher amounts of polyphenols and anthocyanins present in those pigmented rice varieties [4]. Later, we reported on how hot air drying and FIR drying affected the bioactive compounds, as well as antioxidant activity in three varieties of pigmented rice. We found that FIR had shown remarkably positive effects on those parameters that were tested [13]. According to our data, it is doubtful whether these results would be consistent during different years of production (climate changes). Although changes due to FIR drying on bioactive compounds and antioxidant activities were reported in some varieties, there have been some pigmented rice varieties not yet studied. Furthermore, the effects of FIR drying methods on starch digestibility have not been reported elsewhere on these varieties of Thai pigmented rice.

This present study aimed to explore the effects of FIR drying methods on starch digestibility and bioactive compounds, namely, phenolics, flavonoids and anthocyanins, along with the antioxidant activities of Thai rice varieties with red and purple pericarp coloring as well as non-pigmented rice. Furthermore, the comparison contrasted unheated and heated grain, thus to providing useful data for food and agricultural processing, engineering, industrial practice and the incorporation of functional food materials into human diets with health-promoting benefits.

## 2. Materials and Methods

### 2.1. Chemicals and Reagents

Standards of phenolic acids were used, namely ferulic, *p*-coumaric, sinapic, gallic, caffeic, syringic, protocatechuic, vanillic and chlorogenic acids. Standards of flavonoids were used, namely apigenin, quercetin, myricetin and rutin. Standards of anthocyanins were used, namely malvidin, pelargonidin and cyanidin 3-glucoside. All of the standards were purchased from the Sigma–Aldrich Co. (St. Louis, MO, USA). All other solvents and chemicals used were analytical reagent grade.

### 2.2. Sample Preparation

Six rice varieties, Hom Mali or KDML 105 (non-pigmented rice); Sung Yod, Mun Poo, Mali Dang (red rice); and Hom Nil and Riceberry (purple rice), were collected from fields located in Thailand, during the 2018–2019 growing season. Each paddy of pigmented rice was de-husked to provide brown rice. Once obtained, the FIR-dried rice was prepared by FIR and combined with hot air (HA), according to our previous study [4]. The dryer consists of a stainless-steel drying chamber (30 cm × 51 cm × 50 cm), a sample tray (25.4 cm × 37 cm), a centrifugal fan and a far-infrared heater (122 mm × 60 mm). Two sets of three-FIR heaters were placed, one at the bottom and another one at the top of the drying chamber. The sample tray was set midway between, and parallel to, the top and bottom heaters. The hot air was circulated through the drying chamber with a fan. The temperature of inlet air flowing through a hot-air heater was controller with a PID controller (accuracy of ±1 °C). The de-husked rice (10 g) was dried in a drying chamber and then treated with FIR at an air velocity of 1.5 m/s, 40 °C and an intensity of 2 kW/m^2^, 250 W for 2 h. The moisture content (wet basis) of obtained samples were in a range of 12–14%. Samples were ground into a fine powder and then sieved (using an 80 mesh sieve). The FIR treatment was conducted in triplicate. Subsequently, both unheated and FIR-treated rice powder were kept at 4 °C in three different bags before further analysis.

### 2.3. Determination of Total Phenolic Content

The total phenolic content (TPC) was determined according to the method described by Ratseewo et al. [22], using the Folin–Ciocalteu method with gallic acid as a standard. The absorbance of samples was read at 725 nm using a spectrophotometer (Beckman Coulter, Fullerton, CA, USA). The quantity of total phenolics was expressed as mg gallic acid equivalents per 100 g of dry weight (mg GAE/100 g DW).

### 2.4. Determination of Total Flavonoid Content

The total flavonoid content (TFC) was determined using the method of Ratseewo et al. [13], by a colorimetry method. The absorbance of samples was read at 510 nm using a spectrophotometer. The content of flavonoid was expressed as mg rutin equivalents per 100 g dry weight (mg RE/100 g DW).

### 2.5. Determination of Total Anthocyanin Content

The total anthocyanin content (TAC) was determined spectrophotometrically according to Ratseewo et al. [4]. The absorbance was read at 534 nm using a spectrophotometer. The value of total anthocyanin was determined as mg cyanidin equivalents per 100 g dry weight (mg CyGE/100 g DW).

### 2.6. Determination of Antioxidant Activity Using DPPH Radical-Scavenging Activity

The determination of antioxidant activity was involved the 2,2-diphenyl-1-picrylhydrazyl (DPPH) radical scavenging activity assay [13]. The absorbance was determined at 517 nm using a spectrophotometer. All analyses were done in triplicate.

The calculation of DPPH radical scavenging activity as percent inhibition was performed using this following formula:

(%) = [1 − (A(sample) − A(control))] × 100.(1)

### 2.7. Amylose Content

The determination of amylose content involved the iodine dye-binding method according to Knutson [23] with some modification. The samples were weighed (each 5 g) and were mixed with 6 mM iodine and 90% (*v/v*) dimethyl sulfoxide (DMSO) in distilled water (10 mL). Rice samples were incubated at room temperature overnight and shaken at a 180° angle and 20 rpm. After 30 min, the samples were determined using a spectrophotometer at 600 nm (Libra 550, Biochrom, Holliston, MA, USA). The percentages of apparent amylose and amylopectin contents were calculated using an amylose standard from potato by the equation:

(2) $$\%\;\mathrm{Amylose}=\frac{\%\;\mathrm{Apparent}\;\mathrm{amylose}\;-6.2}{93.8}$$

### 2.8. Identification of Phenolic Compounds

The authentic phenolic acids were identified using an HPLC (high performance liquid chromatography) system (Shimadzu LC-20 AC pumps). Phenolic acids were separated on a C18 column (4.6 mm × 250 mm) with a particle size of 5 µm (Hichrom Limited, Berks, UK) and were detected at 280 nm. The HPLC analysis used a diode array detection (SPD-M20 A, DAD), as described by Ratseewo et al. [4]. The quantity of individual phenolic acids was calculated as external standards curves.

### 2.9. Identification of Flavonoids

The authentic individual flavonoids were identified using an HPLC (high performance liquid chromatography) system (Shimadzu LC-20 AC pumps). The individual flavonoids were separated on a C18 column (4.6 mm × 250 mm) with a particle size of 5 µm (Hichrom Limited, Berks, UK) and were detected at 370 nm. The HPLC analysis used diode array detection (SPD-M20 A, DAD), using the same equipment as for phenolic acids. The gradient elution of HPLC system was described by Ratseewo et al. [4].

### 2.10. Identification of Anthocyanins

The analysis of anthocyanins was performed by an RP-HPLC method, using the same equipment as for phenolic acids and flavonoids. The extraction and a gradient elution procedure were described by Ratseewo et al. [4]. The UV-array detector was set at 520 nm. Anthocyanins were quantified by comparing them with their authentic standards curves. The average peak areas were used in calculations.

### 2.11. Differential Scanning Calorimetry (DSC)

Differential scanning calorimetry (DSC) analysis was used to determine the thermal behavior of the rice-starch samples using a Multi-Cell DSC (TA Instruments, Elstree, UK), as described by Ratseewo et al. [13]. Pigmented rice flour materials were extracted to obtain rice starch [13] prior to DSC analysis. The parameters, namely onset temperature (T_o_), peak temperature (T_p_), and conclusion temperature (T_c_) including enthalpy of gelatinization Δ_gel_H (J_g−1_), were recorded from each thermogram of the samples.

### 2.12. Starch Digestion

The kinetics of in vitro starch digestion were studied according to the method of Ratseewo et al. [13] and Warren et al. [24]. To start the starch hydrolysis, α-amylase (80 units in PBS, pH 7.4, Sigma-Aldrich) in 400 μL was added to each rice powder. The starch hydrolysis was monitored by taking sub-samples at pre-defined time points from 1 to 60 min (at 0, 1, 2, 3, 4, 5, 7, 10, 13, 15, 20, 45 and 60 min). The starch hydrolysis products were boiled at 100 °C in a water bath for 5 min and were determined using reducing sugar analysis the 4-hydroxybenzoic acid hydrazide (PAHBAH) reducing sugar assay. The analysis of maltose, the amylolysis product, was performed at an absorbance of 405 nm.

The estimate of the kinetics of in vitro starch digestion was calculated by the first-order equation [25]:

(3) $$C_{t}=C_{\infty }\left(1-e^{-kt}\right)$$

In this equation, the content of maltose (starch digest product) at time *t* was *C_t_*; the equivalent content of maltose (starch digest product) at the end point of the reaction was *C_∞_*; the constant of digestibility rate was *k*. These values were obtained using logarithm of slope (LOS) analysis, as described previously [24,26].

(4) $$\ln\left(\frac{dC}{dt}\right)=\ln\left(C_{\infty }k\right)-kt$$

In addition, a single reaction which is the situation where multiple first-order rate constants are observed can be described by this equation [27]:

(5) $$C_{t}=\{\begin{array}{c} C_{1\infty }\left(1-e^{-k_{1}t}\right)if\;t\leq t_{int}\\ C_{int}+C_{2\infty }\left(1-e^{-k_{2}\left(t-t_{int}\right)}\right)if\;t\geq t_{int} \end{array}$$

This formula exhibited the parameter, namely, the time of intersection of the two plots was *t_int_* and the content of product at *t_int_* was *C_int_*. *C*_1∞_ was the end point of the reaction of single phase or first phase. *C*_2∞_ was the end point of the reaction of second phase. *k*_1_ and *k*_2_ were the first- and second-rate constants of each phase. Total *C_∞_* is the sum of *C*_1∞_ and *C*_2∞_ and represents the total extent of starch amylolysis.

### 2.13. Statistical Analysis

The data are expressed as the mean ± standard deviation (SD). Analysis of variance (ANOVA) was analyzed using the statistical analysis system (SPSS 11.5 for Windows; SPSS Inc., Chicago, IL, USA). All of the experiments were tested in triplicate. Duncan’s multiple range tests were analyzed. The confidence limits used in this study were based on 95% (*p* < 0.05).

## 3. Results and Discussion

### 3.1. Total Phenolic Content (TPC)

**Figure 1:**
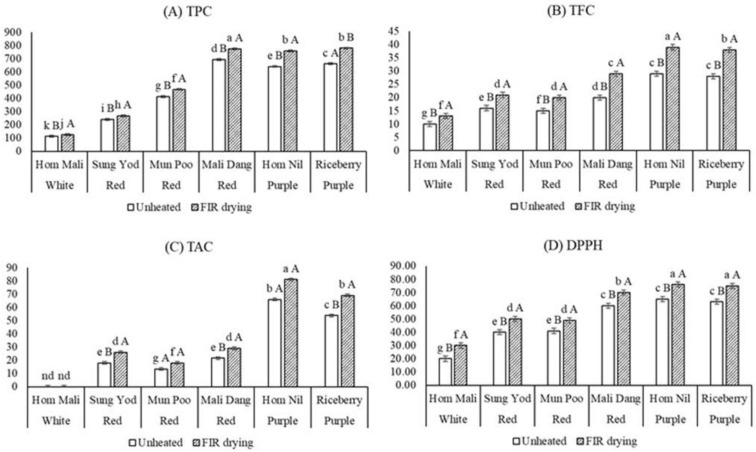
Effect of FIR drying on (**A**) total phenolic content; TPC (mg GAE/100 g DW), (**B**) total flavonoid content; TFC (mg RE/100 g DW), (**C**) total anthocyanin content (TAC) (mg CyGE/100 g) and (**D**) antioxidant capacity (DPPH) (%) of pigmented rice. Values are expressed as mean ± standard deviation (*n* = 3). Means with different capital letters of unheated and FIR drying rice in the same rice variety as affected by different drying methods were significantly different at the level (*p* < 0.05). Lowercase superscripts are used for comparison of all samples.

The total phenolic content of unheated pigmented rice ranged from 112 mg GAE/100 g dw in KDML105 (non-pigmented rice) to 680 mg GAE/100 g dw in Mali dang (Figure 1A). The total phenolic content was significantly different among the rice samples studied. All pigmented rice samples had higher TPC than white rice. Similarly, a range of results for TPC in pigmented rice was observed in rice from different growth sites, i.e., China, Sri Lanka, Thailand [13,28], and Australia [2]. FIR-dried samples had higher TPC than unheated rice, ranging from 10% (KDML 105) to 16% (Hom Nil). Similar findings were reported in many research reports. The amount of TPC increased after drying by FIR in all six rice varieties, with increases from 7.5% to 18% in pigmented rice [4] and 25% in soy-milk residue extracts [21], 8% in marigold flowers [29], 5% in Kaprow leaves [19] and a dramatic increase (10 times) in infrared-dried strawberry [30].

The explanation of these phenomena could be that the heat generated by FIR via molecular vibrations is absorbed rapidly and steadily to the centre of the rice grains evenly during drying. This treatment may result in the breaking down of the covalent bonds. Hence, FIR heating could release and activate the small-molecule phenolic compounds in biomaterials [18,20] which would increase the TPC of dried pigmented rice in our present study. In addition, phenolic groups could increase after thermal processing. Possibly these phenolic compounds could be liberated from wall structures and detached from other macro molecules such as proteins, fibers and pectins via hydrolysis, due to the breaking of covalent bonds [31]. However, in some cases, thermal instability may cause an increase or decrease of total phenolics [9]. We speculate that changes in the internal structures and compounds in pigmented rice were significantly affected by FIR treatment, due to increases in extractable matter, resulting in increased extraction yields of phenolics and other components.

### 3.2. Total Flavonoid Content (TFC)

The total flavonoid contents of the six rice varieties ranged from 10.01 mg RE/g DW in KDML 105 to 39.12 mg RE/g DW in Hom Nil (Figure 1B). TFC of FIR-treated rice to different thermal processing was significantly (*p* < 0.05) higher than that of unheated pigmented rice. The content of total flavonoids increased by approximately 1.3–1.4-fold in FIR-dried rice, compared with unheated rice. The TFC of rice grains seemed to increase when irradiation of far infrared, combined with hot air, was applied. Similarly, to trends in the results of TPC, TFC was enhanced by FIR drying and the proposed reasons could be explained as the same with that of phenolic compounds. Generally, flavonoids are a group of polyphenol secondary metabolites which have many functions including being natural antioxidants in plant materials [18]. The irradiation by far infrared may vibrate covalent bonds, causing the release of lower molecular weight phenolics and flavonoid compounds from the matrix and structure of rice [13]. Many studies have also reported an increase in the TFC of heated plants, such as Thai rice [13], grape seeds [32] and peanut hulls [20], due to the liberation of flavonoid compounds and some other phenolics.

### 3.3. The Total Anthocyanin Content (TAC)

The TAC of pigmented rice varieties at different thermal treatments are presented in Figure 1C. The TAC of all unheated samples ranged from non-detected in normal rice (KDML 105) to 69 mg/100 g dw in purple rice (Hom Nil). Similar results were shown for TAC values of those varieties, but higher amounts were found in FIR-treated rice ranging from 0–81 mg/100 g. The highest concentration of total anthocyanin was observed in purple rice, followed by red-rice samples. A previous study reported that untreated black/purple rice from Thailand and China showed higher TAC values than red-rice varieties [28]. Our results in this study were similar to the previous study [28]. After FIR treatment, all six rice varieties showed significant increases in the TAC (*p* < 0.05). FIR-dried rice may increase TAC that are similar to TPC and TFC. Similar results were observed by the increases in anthocyanins after the FIR treatment of pigmented rice [4], including cyanidin-3-glucoside, one of the major anthocyanins of pigmented rice, which may account for increases in the total content of anthocyanins in this research. Our findings were consistent with a previous report [33], that thermal treatment commonly induced an increase in the main phenolic compounds such as the anthocyanins and total cinnamates in orange juice.

### 3.4. Antioxidant Activity

The DPPH radical scavenging activity of the unheated and FIR-treated pigmented and non-pigmented rice varieties is presented in Figure 1D. Both red and purple samples had higher values of inhibition (%) than white rice. Similar trends were observed in TPC, TFC and TAC, depending on the pericarp color. A similar result was reported in previous studies [2,4,34]. The inhibition (%) of DPPH radical pigmented rice depends on the pericarp of the grains, especially the pigmentation of the pericarp. After dried rice, the highest values of DPPH radical scavenging activity were observed in all FIR-dried samples when compared to unheated samples. The results indicated FIR radiation increased TPC, TFC, TAC and antioxidant activity. Similar results were detected for the antioxidant activity which increased after FIR treatments of rice bran (increased by 20%) and rice husk (increased by 35%) [18], tomato (21%) and papaya (21%) [35]. In addition, Adak et al. [30] reported a massive increase of antioxidant activity in strawberry as affected by infrared drying (1.5 m·s^−^^1^, 200 W, 100 °C). Generally, most natural antioxidants are bound in covalent form to insoluble polymers. When FIR drying is applied to the biomaterial containing those compounds, the radiation of far-infrared is able to activate and release low molecular-weight natural antioxidants, such as phenolic acids, flavonoids, etc., in samples if this bonding is attacked [20].

### 3.5. Phenolic Compounds

The individual phenolic compounds of unheated and FIR-treated rice samples are shown in Table 1. The predominant phenolic acids found in non-pigmented rice included FA, p-CA and PCCA whereas those compounds, along with VA, were found in all pigmented varieties studied, like the results reported for Australia rice [2].

These results have been consistent with the data from previous research in different years of production that the major phenolic acid in non-pigmented and pigmented rice types (Hom Nil and Riceberry), is FA. It is normally present in the rice bran layers, which may be derived from arabinoxylans, pectin and/or cross-linked to polysaccharides in cell walls [36]. All rice samples treated with FIR had greater phenolic acid contents than untreated rice. GA, FA and SNA were significantly (*p* < 0.05) increased by FIR in all treated samples. Similarly, previous research reported that ChA and p-CA acids of tomato and papaya were significantly increased by FIR treatment [35].

### 3.6. Composition of Flavonoids

When compared between FIR and untreated rice, the highest values of quercetin were found in FIR-dried samples of the six rice varieties, ranging from 5.43 to 23.94 µg/100 g (Table 2). Rutin and myricetin were significantly reduced (*p* < 0.05) by FIR treatment in all pigmented rice varieties, while apigenin and quercetin were significantly increased (*p* < 0.05) when compared to unheated samples. Apigenin and quercetin contents were increased significantly in all purple rice samples, by 18% and 41%, as affected by FIR, respectively.

This result agrees with our previous study of three varieties of Thai pigmented rice harvested in the previous year [13]; however, a slightly smaller increase was observed. In addition, FIR treatment has been reported to enhance some flavonoids in other food materials: for example, increased myricetin in papaya (1.2 times); quercetin (20.2 times), apigenin in tomato (3.18 times) [35] and quercetin (4.7 times) in buckwheat sprouts [37]. According to the results obtained from our present study, the explanation of increases in quercetin and apigenin accumulation may be caused by the rupture of the glycoside bonds of rutin by FIR [35]. Rutin has a chemical structure similar to that of quercetin, however, it has an extra glycone flavonoid, whereas quercetin does not. A further consideration is that rutin (glycoside bonds) was thought to be the hydrolysis pathway to quercitin (aglycoside bonds) [38]. Previous research [4,20,35] has also explained that the covalent bonds of polymerized polyphenols are broken, thus causing the alteration of HMW (high-molecular weight) to LMW (low-molecular weight) compounds.

### 3.7. Composition of Anthocyanin

Table 2 classified the individual anthocyanin in different rice varieties studied, compared with the authentic standards. The results showed that anthocyanins were absent in non-pigmented rice (KDML 105), while all three anthocyanin derivatives were found in pigmented rice varieties studied. Cyanidin 3-glucoside was higher in purple rice varieties than others. A previous study by Abdel-Aal et al. [6] reported similar results for Indian rice varieties, namely that anthocyanins (particularly cyanidin-3-O-glucoside) were found to the greatest extent in purple and black rice cultivars. The different anthocyanin contents of the various rice varieties may be caused by genetic or environmental effects. They also found that malvidin was present at a high content in all red varieties [6,7]. We previously reported on how FIR treatment affected the anthocyanins in three pigmented rice varieties: Mali dang, Hom Nil and Riceberry [13] and found that malvidin, cyanidin-3-glucosides and pelargonidin increased after FIR treatment. When compared to our findings in this present study with the same varieties but in a different production year and for two more varieties (Mun Poo and Sung Yod), the results were comparable. The explanation of this occurrence affected by FIR treatment may be similar to that in phenolic acid and flavonoids. Scalzo et al. stated that thermal treatment generally induced an increase in the main phenolic substances of orange juice, such as the anthocyanins and total cinnamates [33].

### 3.8. Amylose Content

Amylose content is one of the most significant parameters indicating the quality of rice, because it affects the cooking quality [39]. All red and purple rice varieties had the highest amylose contents; while KDML105, a non-pigmented variety, had the lowest content (Table 3). The amylose content of all pigmented rice varieties was not significantly different when compared to unheated rice. Several studies have determined the content of amylose in different Thai rice varieties and the values ranged from 2% in white to 19% in purple rice [12,13,40]. 

The contents of amylose that were in the range between 10 and 20% are classified as a low-amylose-content rice [41]. According to the data obtained from our present study, all varieties are classified as low-amylose types (below 20%). In general, typical Thai rice contains low-amylose content, thus providing a relatively soft and adhesive texture after cooking [42]. Therefore, this research suggested that different reactions to FIR drying do not relate to amylose content in pigmented rice varieties.

### 3.9. Differential Scanning Calorimetry (DSC)

Differential scanning calorimetry (DSC) has demonstrated different values in onset (T_o_), peak (T_p_) and concluding temperatures (T_c_) and gelatinization enthalpy (Δ_gel_H) parameters for extracted samples of rice starch (Table 3). The onset, peak and conclusion temperatures (T_o_, T_p_ and T_c_) of unheated rice starch ranged from 58.04–74.18 °C to 65.75–79.18 °C and 73.82–82.90 °C. For FIR-treated samples, those parameters were in ranges of 58.08–73.73 °C, 65.64–78.81 °C and 73.38–82.72 °C, respectively. The Δ_gel_H of unheated rice ranged from 9.55 in Mun Poo (red) to 12.55 in Sung yod (red); among those, the Δ_gel_H of KDML105 was not significantly different with Mun Poo. After FIR treatment, there was a significant increase in Δ_gel_H, with Sung yod (13.54 J g^−1^), Mali Dang (12.44 J g^−1^) and Riceberry (11. 74 J g^−1^) having greater values of Δ_gel_H than any other rice variety. However, the gelatinization temperatures, T_o_ and T_p_ of Sung Yod as treated by FIR were slightly decreased when compared to unheated samples (74.18 to 73.73 °C) while, the reducing of T_c_ was observed in Ricberry from 73.82 to 73.38 °C (*p* < 0.05). Sung Yod was also observed with the highest temperatures for T_o_, T_p_ and T_c_.

The relationships between the gelatinization parameters including T_o_, T_p_, and T_c_ and the internal crystalline structure of starch and its heat stability, as well as the ∆H reflects the energy needed for double helical dissociation and hence the crystallinity of starch [43,44]. Gelatinization behavior may be influenced by starch characteristics; however, the starches chosen for our present study were comparatively similar in many aspects. In this study, the rice starch was extracted from rice flour for investigation only of starch behavior as affected by FIR. The starches of pigmented rice had similar Δ_gel_H (J_g−1_) though they had higher gelatinization temperatures. As can be observed, FIR-treated pigmented-rice varieties were found to have greater gelatinization enthalpy values, compared with unheated samples. Therefore, this research suggested that FIR drying gave greater effects on gelatinization behavior in pigmented rice varieties than normal rice.

### 3.10. In Vitro Digestibility

The starch digestibility of pigmented rice cultivars, as affected by FIR treatment, are presented in Table 4. The values of digested starch (60 min) in unheated rice ranged from 9.57–16.17% while digested starch content in FIR-treated rice ranged from 7.33–12.17%. The values of digested starch (%) in unheated rice were greater in the following order: KDML 105 > Mun Poo > Mali Dang and Riceberry > Hom Nil > Sung Yod (*p* < 0.05). In the case of FIR-treated rice, those values were significantly lower in the following order: KDML 105 (non-pigmented rice) > Mun Poo > Sung Yod, Mali Dang, Riceberry and Hom Nil. We characterized the digestion of the rice samples as low rates during the digestion process at small intestine (approximately after 60 min), as shown in Table 4. The rice digestibility was determined using single and two phases by plotting LOS analysis of each sample. For the single phase of samples, the value of k_1_ (the digestibility rate constant) for pigmented rice (Mun Poo, red rice) and non-pigmented rice (KDML105) being treated by FIR were substantially lower compared to unheated rice. This reflects their slower digestion rates by FIR treatment, with values ranging from 0.028 to 0.041 min^−^^1^ in Mun Poo and from 0.028 to 0.071 min^−^^1^ in KDML 105. In the present study, the normal rice exhibited the highest of C_∞_ value (17.11%). When considering the digestion kinetics, Hom Nil, Mali Dang, Riceberry, and Sung Yod could be the most suitable as modelled by performing first order rate constants twice [27]. As a result, the estimated values of k (rate of starch digestion) and C_∞_ (extent of amylolysis) for variables in Equation (3) for those samples are shown in Table 4. According to observations, when the starch digestion occurred in two phases, this indicates a slower phase in digestion. The k_2_ values of unheated rice samples varieties were found highest in Mali Dang, followed by Hom Nil, Riceberry and Sung Yod, respectively. These values can be compared to k_1_ values, where amylolysis occurs as a single-phase process in a certain sample. In the case of FIR-dried samples, the second rate (k_2_) values were found to be the highest in Mali Dang (0.052), followed by Riceberry (0.041), Hom Nil (0.039) and Sung Yod (0.030). 

The C_∞_ (extent of amylolysis) decreased similarly in FIR-treated rice when compared to unheated rice. The first-order kinetic data explains the amylolysis of starch in rice from white, red and purple rice varieties, as are explained in this paper. The values (C_∞_ and k) of all parameters related to starch digestion (in vitro) were significantly lower for all FIR-treated pigmented samples, when compared to unheated rice. Although the results of enthalpy in both unheated and FIR-treated samples showed the highest values in Sung Yod varieties, the percentages of starch digested were lower for the purple-rice varieties (Hom Nil and Riceberry). In addition, the results of DSC of unheated and FIR-dried rice samples (Table 3) also showed the lower values of gelatinization enthalpy for normal rice. The gelatinization enthalpy increased in all samples after FIR treatment. DSC analysis was used for rice starch determination, hence the influence of starch structure for any variety of rice was a significant factor of starch digestibility. In this present study, the normal or non-pigmented rice sample (KDML105) had the highest contents of digestible starch in both unheated and FIR-treated form. When compared with the other rice varieties studied, the KDML105, as a non-pigmented rice, contains other phenolic compounds, although it has no anthocyanins. This may cause the higher digestibility of the KDML105 relative to the pigmented varieties. These results indicated that starch digestibility in pigmented rice could be associated with phenolic compounds and anthocyanins. Anthocyanins have been exhibited to possess in vitro inhibition of *α*-glucosidase activity. Previous studies reported significant activity of *α*-glucosidase inhibition of polyphenols and anthocyanins from pigmented potato extract and these compounds were able to reduce starch digestibility [16,17]. Moreover, many studies have shown that the main anthocyanins in pigmented rice were peonidin 3-glucoside and cyanidin 3-glucoside, while these compounds were absent in non-pigmented or white rice [12,28,45]. Among anthocyanin derivatives, the most effective inhibitor of *α*-glucosidase activity is cyanidin 3-glucoside [46], a phenolic compound in millet extracts that could inhibit both enzymes *α*-amylase and *α*-glucosidase. Additionally, Tadera et al. [47] have reported flavonoid compounds (namely, genistein, daidzein, kaempferol, naringenin and apigenin) as having the capacity of *α*-glucosidase inhibition. Recently, Tangkhawanit et al. [21] have observed that TPC, TFC and flavonoids increased in soymilk residue after FIR treatment and that the extracts were more efficient in inhibiting *α*-amylase when compared with hot air-dried extract; thus, drawing the conclusion of them being involved in lowering starch digestion. In our present study, we found that ferulic acid (FA) is an abundant phenolic acid in rice. FA has been reported to be beneficial in the treatment of type-2 diabetes [48], since it controls blood-glucose levels (BGLs) by increasing glucokinase functions and producing glycogen in the liver. Furthermore, FA significantly lowered BGLs and raised levels of plasma insulin in mice [48]. Our research has suggested that phenolic compounds, flavonoids and anthocyanins may play important roles in decreasing starch amylolysis and the absorption of glucose in the small intestine; therefore, they may play a possible role in preventing diabetes by controlling post-prandial glycaemia. According to our data, it could be stated that phenolic compounds have the capacity to combine with an amino group in protein or glycoside bonds in conjugated polysaccharides by covalent and non-covalent bonds, thus inactivating a digestive enzyme [21].

The results of our present study may support the literature reports that bioactive compounds in rice, such as ferulic acid, protocatechuic acid, quercetin, cyanidin 3-glucoside, apigenin and malvidin, may play an important role in inhibiting *α*-amylase activity in pigmented rice. Thus, our present study has established that starch structure, along with bioactive compounds of pigmented rice, may have an impact on lower digestibility. Furthermore, heat treatment using FIR could be a new factor to assist in aiding the extractable yield of those compound which may be associated with greater capacity for retarding the starch-digestion rate of pigmented rice. Consumption of slowly digested rice starches is related to a reduced risk of developing type-2 diabetes and cardiovascular disease. Accordingly, our discoveries have offered valuable data for the development of slowly digested starch of biomaterials for application in the practice of providing nutritional options for individuals with metabolic disorders such as type-2 diabetes and obesity, via the use of drying processing such as FIR.

## 4. Conclusions

Our results have indicated that FIR provided both negative and positive impacts of FIR treatment on starch digestion and bioactive compounds of pigmented rice and normal rice. For a negative impact, FIR reduced the content of certain heat-sensitive substances such as sinapic acid and rutin. On the other hand, the application of FIR increased the levels of total phenolic, flavonoid and anthocyanin contents in all rice samples. However, the changes of individual compounds such as phenolic acids and flavonoids were varied. FIR increased the functional properties of antioxidants by scavenging DPPH-radical reducing power, capable of inhibiting α-amylase, thereby lowering the starch digestibility of rice. These data indicate that the reduction of the starch digestion rate may result from the association of starch structure and the inhibitory effects of polyphenols in pigmented rice.

This study suggests that FIR may have a favorable effect on extraction yield for bioactive compounds of the grain by assisting the release of small or free molecules from the complex compounds. Hence, the selection of an appropriate drying method could be a smart way for enhancing the functional properties and nutritional quality of rice, thus contributing to consumer health benefits. In addition, our results have indicated that the lower gelatinization enthalpy and higher starch digestibility of white or non-pigmented rice varieties, when compared with those of pigmented rice, could be affected by the absence of anthocyanins, even though it contains a comparable number of polyphenols and flavonoids. However, white or non-pigmented rice may have more desirable sensory quality (e.g., being stickier). Our results suggest that Hom Nil (purple rice) may be the best variety for drying with FIR treatment for future works because Hom Nil was enhanced the most of cyanidin 3-glucoside (67%) by FIR, which led to a decreasing digestibility rate constant (32%) and less starch digested (37%). This research delivers important indications that FIR could be considered as an appropriate drying method for pigmented rice regarding health benefits. Our findings are expected to be supportive for applications to the food, nutraceutical and pharmaceutical industries.

## Figures and Tables

**Table 1:** Identifications of phenolic acids in pigmented rice varieties as affected by FIR.

Rice Varieties	Treatments	Phenolic Acids (µg/100 g)									
		GA	PCCA	VA	ChA	CFA	SyA	*p*-CA	FA	SNA	Total
KDML105	Unheated	2.42 ± 0.07 ^f^	14.48 ± 0.56 ^d^	2.24 ± 0.08 ^g^	2.66 ± 0.12 ^c^	1.45 ± 0.04 ^c^	1.53 ± 0.09 ^f^	3.32 ± 0.26 ^h^	16.50 ± 0.63 ^e^	1.14 ± 0.06 ^j^	45.75 ± 2.42 ^e^
(White)	FIR	2.98 ± 0.11 ^e^	21.12 ± 0.11 ^a^	2.49 ± 0.09 ^g^	2.99 ± 0.19 ^ab^	1.46 ± 0.01 ^c^	1.66 ± 0.04 ^ef^	3.55 ± 0.09 ^h^	22.12 ± 0.87 ^c^	1.21 ± 0.08 ^i^	59.58 ± 2.52 ^c^
Sung Yod	Unheated	2.35 ± 0.07 ^f^	13.58 ± 0.19 ^e^	3.49 ± 0.07 ^f^	1.41 ± 0.09 ^e^	1.26 ± 0.07 ^d^	1.51 ± 0.05 ^f^	3.72 ± 0.17 ^g^	16.85 ± 0.21 ^e^	1.49 ± 0.04 ^h^	45.66 ± 3.45
(Red)	FIR	2.97 ± 0.13 ^e^	20.32 ± 0.13 ^b^	5.81 ± 0.21 ^c^	1.45 ± 0.12 ^de^	1.29 ± 0.03 ^d^	1.74 ± 0.06 ^de^	3.98 ± 0.07 ^f^	22.33 ± 1.43 ^c^	1.62 ± 0.05 ^g^	61.51 ± 4.58 ^c^
Mun Poo	Unheated	2.29 ± 0.25 ^f^	12.64 ± 0.67 ^f^	3.74 ± 0.12 ^e^	2.52 ± 0.05 ^c^	1.46 ± 0.05 ^bc^	1.55 ± 0.07 ^e^	5.51 ± 0.22 ^e^	16.83 ± 0.15 ^e^	1.50 ± 0.09 ^h^	48.04 ± 3.71 ^e^
(Red)	FIR	2.97 ± 0.14 ^e^	18.38 ± 0.12 ^d^	4.24 ± 0.22 ^d^	2.61 ± 0.11 ^c^	1.44 ± 0.02 ^c^	1.75 ± 0.05 ^d^	6.12 ± 0.12 ^d^	23.57 ± 1.22 ^c^	1.65 ± 0.04 ^g^	62.35 ± 4.79 ^c^
Mali Dang	Unheated	3.31 ± 0.11 ^d^	13.21 ± 0.26 ^ef^	3.76 ± 0.09 ^e^	1.50 ± 0.07 ^de^	1.54 ± 0.05 ^b^	1.63 ± 0.05 ^e^	5.43 ± 0.23 ^e^	17.71 ± 0.26 ^d^	2.32 ± 0.05 ^f^	50.42 ± 4.91 ^d^
(Red)	FIR	4.98 ± 0.11 ^c^	18.87 ± 0.13 ^c^	4.31 ± 0.18 ^d^	1.68 ± 0.11 ^d^	1.58 ± 0.05 ^b^	1.89 ± 0.01 ^c^	5.77 ± 0.11 ^e^	22.65 ± 1.27 ^c^	2.61 ± 0.18 ^e^	64.34 ± 4.31 ^c^
Hom Nil	Unheated	6.29 ± 0.15 ^b^	14.82 ± 0.72 ^d^	6.71 ± 0.06 ^b^	2.75 ± 0.08 ^bc^	1.71 ± 0.06 ^a^	1.74 ± 0.08 ^d^	6.58 ± 0.23 ^c^	33.11 ± 0.42 ^b^	5.01 ± 0.17 ^d^	78.72 ± 4.28 ^b^
(Purple)	FIR	6.96 ± 0.21 ^a^	20.98 ± 0.15 ^a^	7.01 ± 0.31 ^a^	3.21 ± 0.15 ^a^	1.72 ± 0.09 ^a^	1.99 ± 0.01 ^b^	6.81 ± 0.21 ^c^	40.22 ± 1.35 ^a^	5.32 ± 0.11 ^c^	94.22 ± 4.71 ^a^
Riceberry	Unheated	6.24 ± 0.09 ^b^	14.76 ± 0.34 ^d^	6.70 ± 0.08 ^b^	2.76 ± 0.04 ^bc^	1.76 ± 0.04 ^a^	1.97 ± 0.05 ^a^	7.21 ± 0.13 ^b^	32.32 ± 0.37 ^b^	5.14 ± 0.26 ^b^	78.76 ± 3.98 ^b^
(Purple)	FIR	6.87 ± 0.16 ^a^	21.07 ± 0.15 ^a^	7.12 ± 0.24 ^a^	3.11 ± 0.16 ^a^	1.76 ± 0.07 ^a^	2.03 ± 0.06 ^a^	7.66 ± 0.21 ^a^	40.28 ± 2.39 ^a^	5.43 ± 0.11 ^a^	95.33 ± 4.64 ^a^

GA (Gallic), PCCA (Protocatechuic), VA (Vanillic), ChA (chlorogenic), CFA (Caffeic), SyA (Syringic), p-CA (p-Coumaric), FA (Ferulic) and SNA (Sinapic) acids. Mean values within a column superscripted by the small letter are significantly different at *p* < 0.05.

**Table 2:** Identifications of flavonoids and anthocyanin in pigmented rice varieties as affected by FIR.

Rice Varieties	Treatments	Flavonoids (µg/100 g)				Anthocyanin (µg/100 g)		
		Rutin	Myricetin	Quercetin	Apigenin	Cyanidin-3-glucoside	Pelargonidin	Malvidin
KDML105	Unheated	2.73 ± 0.04 ^g^	9.43 ± 0.11 ^e^	5.43 ± 0.21 ^e^	2.42 ± 0.06 ^f^	ND	ND	ND
(White)	FIR	2.02 ± 0.07 ^h^	7.87 ± 0.15 ^f^	7.52 ± 0.22 ^d^	2.76 ± 0.04 ^d^	ND	ND	ND
Sung Yod	Unheated	3.72 ± 0.14 ^c^	15.32 ± 0.45 ^c^	9.12 ± 1.08 ^c^	2.44 ± 0.01 ^f^	9.56 ± 1.43 ^g^	0.43 ± 0.04 ^f^	1.36 ± 0.07 ^g^
(Red)	FIR	2.96 ± 0.11 ^f^	10.32 ± 1.17 ^e^	15.37 ± 1.27 ^b^	3.44 ± 0.07 ^c^	20.44 ± 0.75 ^e^	3.43 ± 0.09 ^d^	1.47 ± 0.02 ^e^
Mun Poo	Unheated	3.32 ± 0.09 ^de^	15.32 ± 1.26 ^b^	10.48 ± 1.68 ^c^	2.54 ± 0.03 ^e^	9.55 ± 1.48 ^g^	0.24 ± 0.02 ^g^	1.43 ± 0.08 ^f^
(Red)	FIR	2.76 ± 0.08 ^g^	9.91 ± 1.18 ^e^	15.21 ± 1.82 ^b^	3.64 ± 0.11 ^c^	18.31 ± 0.33 ^f^	3.21 ± 0.01 ^e^	1.58 ± 0.05 ^d^
Mali Dang	Unheated	4.38 ± 0.21 ^b^	17.55 ± 1.76 ^b^	14.38 ± 1.96 ^b^	2.49 ± 0.09 ^ef^	10.45 ± 1.32 ^g^	0.45 ± 0.02 ^f^	1.52 ± 0.04 ^d^
(Red)	FIR	3.21 ± 0.02 ^e^	12.43 ± 1.64 ^de^	23.43 ± 1.39 ^a^	3.54 ± 0.08 ^c^	25.76 ± 0.04 ^d^	4.11 ± 0.07 ^c^	1.73 ± 0.09 ^c^
Hom Nil	Unheated	5.51 ± 0.02 ^a^	22.32 ± 2.11 ^a^	15.34 ± 1.36 ^b^	4.54 ± 0.10 ^b^	41.56 ± 3.87 ^c^	2.36 ± 0.13 ^b^	3.62 ± 0.12 ^b^
(Purple)	FIR	3.41 ± 0.04 ^d^	15.94 ± 1.15 ^b^	23.96 ± 1.32 ^a^	5.42 ± 0.11 a	127.70 ± 0.13 ^a^	6.32 ± 0.27 ^a^	4.87 ± 0.14 ^a^
Riceberry	Unheated	5.43 ± 0.13 ^a^	20.31 ± 2.09 ^a^	14.02 ± 2.33 ^b^	4.43 ± 0.05 ^b^	37.29 ± 2.94 ^c^	2.46 ± 0.09 ^b^	3.78 ± 0.29 ^b^
(Purple)	FIR	3.32 ± 0.17 ^de^	14.66 ± 1.07 ^b^	23.94 ± 2.72 ^a^	5.46 ± 0.14 ^a^	61.71 ± 0.14 ^b^	5.87 ± 0.39 ^a^	4.85 ± 0.11 ^a^

Mean values within a column superscripted by the small letter are significantly different at *p* < 0.05. ND is not detected.

**Table 3:** Gelatinization parameters of pigmented rice varieties as affected by FIR.

Samples	Treatments	% Amylose Content	To (°C)	Tp ( °C)	Tc ( °C)	Δ_gel_H (J g^−1^ Starch)
KDML105	Unheated	12.36 ± 0.12 ^d^	61.19 ± 0.12 ^e^	67.88 ± 0.11 ^e^	74.11 ± 0.10 ^d^	9.63 ± 0.36 ^g^
(White)	FIR	12.45 ± 0.11 ^d^	61.12 ± 0.05 ^e^	67.64 ± 0.07 ^e^	74.10 ± 0.07 ^d^	10.33 ± 0.21 ^ef^
Sung Yod	Unheated	14.42 ± 0.13 ^bc^	74.18 ± 0.08 ^a^	79.18 ± 0.09 ^a^	82.90 ± 0.23 ^a^	12.55 ± 0.42 ^b^
(Red)	FIR	14.55 ± 0.12 ^b^	73.73 ± 0.05 ^b^	78.81 ± 0.05 ^b^	82.72 ± 0.11 ^a^	13.54 ± 0.02 ^a^
Mun Poo	Unheated	15.77 ± 0.18 ^a^	62.45 ± 0.15 ^c^	69.09 ± 0.04 ^d^	74.96 ± 0.16 ^c^	9.55 ± 0.39 ^g^
(Red)	FIR	15.75 ± 0.12 ^a^	62.39 ± 0.19 ^cd^	69.10 ± 0.54 ^cd^	74.90 ± 0.12 ^c^	10.51 ± 0.32 ^e^
Mali Dang	Unheated	15.65 ± 0.14 ^a^	62.21 ± 0.11 ^cd^	68.77 ± 0.11 ^d^	74.95 ± 0.29 ^c^	11.53 ± 0.43 ^d^
(Red)	FIR	15.70 ± 0.14 ^a^	62.20 ± 0.09 ^d^	68.87 ± 0.53 ^d^	74.86 ± 0.24 ^c^	12.44 ± 0.33 ^b^
Hom Nil	Unheated	14.20 ± 0.18 ^c^	60.59 ± 0.11 ^f^	69.54 ± 0.12 ^c^	75.74 ± 0.12 ^b^	9.78 ± 0.76 ^fg^
(Purple)	FIR	14.31 ± 0.15 ^c^	60.43 ± 0.11 ^f^	69.32 ± 0.54 ^cd^	75.70 ± 0.21 ^b^	11.02 ± 0.31 ^d^
Riceberry	Unheated	14.08 ± 0.12 ^c^	58.04 ± 0.08 ^g^	65.75 ± 0.08 ^f^	73.82 ± 0.25 ^d^	10.81 ± 0.39 ^de^
(Purple)	FIR	14.18 ± 0.12 ^c^	58.08 ± 0.11 ^g^	65.64 ± 0.05 ^f^	73.38 ± 0.06 ^e^	11.74 ± 0.28 ^c^

Onset (T_o_), peak (T_p_) and concluding (T_c_) temperatures of gelatinization are shown. Δ_gel_H is the enthalpy change associated with the gelatinization of 1 g of purified starch. Mean values within a column superscripted by the small letter are significantly different at *p* < 0.05.

**Table 4:** The estimate values from LOS plots for all pigmented rice varieties as affected by FIR.

Sample	Treatments	%Starch Digestedat 60 min.	Single or First Phase		Second Phase		Total *C_∞_* (%)
			*C*_1∞_ (%)	*k*_1_ (min^−1^)	*C*_2∞_ (%)	*k*_2_ (min^−1^)	
KDML105	Unheated	16.21 ± 0.19 ^a^	17.21 ± 0.59 ^a^	0.071 ± 0.001 ^f^	N/A	N/A	17.44 ± 0.59 ^a^
(White)	FIR	12.17 ± 0.19 ^c^	12.81 ± 0.59 ^b^	0.059 ± 0.001 ^g^	N/A	N/A	12.81 ± 0.10 ^e^
Sung Yod	Unheated	9.57 ± 0.02 ^d^	3.46 ± 0.11 ^f^	0.198 ± 0.001 ^d^	8.99 ± 0.12 ^b^	0.041 ± 0.002 ^c^	12.45 ± 0.75 ^e^
(Red)	FIR	7.43 ± 0.02 ^f^	2.32 ± 0.11 ^g^	0.185 ± 0.002 ^e^	5.64 ± 0.11 c	0.030 ± 0.001 ^d^	7.96 ± 0.18 ^g^
Mun Poo	Unheated	13.31 ± 0.40 ^b^	13.54 ± 2.23 ^b^	0.057 ± 0.003 ^h^	N/A	N/A	13.54 ± 0.11 ^d^
(Red)	FIR	8.55 ± 0.32 ^e^	8.68 ± 1.11 ^c^	0.044 ± 0.002 ^i^	N/A	N/A	8.68 ± 0.06 ^f^
Mali Dang	Unheated	12.13 ± 1.35 ^bc^	6.00 ± 0.71 ^d^	0.364 ± 0.003 ^a^	9.76 ± 0.15 ^a^	0.065 ± 0.001 ^a^	15.76 ± 0.23 ^b^
(Red)	FIR	7. 54 ± 0.04 ^f^	3.32 ± 0.21 ^f^	0.221 ± 0.003 ^c^	4.57 ± 0.11 ^d^	0.052 ± 0.001 ^b^	7.89 ± 0.11 ^g^
Hom Nil	Unheated	11.69 ± 1.11 ^c^	3.89 ± 0.49 ^f^	0.362 ± 0.002 ^a^	9.57 ± 0.14 ^a^	0.058 ± 0.002 ^b^	13.46 ± 0.13 ^d^
(Purple)	FIR	7.33 ± 0.30 ^f^	3.28 ± 0.29 ^f^	0.211 ± 0.001 ^c^	4.61 ± 0.31 ^d^	0.039 ± 0.001 ^c^	7.79 ± 0.17 ^g^
Riceberry	Unheated	12.50 ± 1.32 ^bc^	4.96 ± 0.07 ^e^	0.313 ± 0.003 ^b^	9.71 ± 0.17 ^a^	0.059 ± 0.001 ^b^	14.67 ± 0.14 ^c^
(Purple)	FIR	7.65 ± 0.81 ^f^	3.34 ± 0.07 ^f^	0.219 ± 0.004 ^c^	4.59 ± 0.07 ^d^	0.041 ± 0.004 ^c^	7.93 ± 0.13 ^g^

The amylolysis occurred by a single-phase process, and that no second phase was observed. Value for the second phase is therefore not applicable (N/A). Mean values within a column superscripted by the same letter are significantly different at *p* < 0.05.

## Data Availability

Data is contained within the article.
